# Design, Synthesis, and Antisickling Investigation of a Nitric Oxide-Releasing Prodrug of 5HMF for the Treatment of Sickle Cell Disease

**DOI:** 10.3390/biom12050696

**Published:** 2022-05-12

**Authors:** Rana T. Alhashimi, Mohini S. Ghatge, Akua K. Donkor, Tanvi M. Deshpande, Nancy Anabaraonye, Dina Alramadhani, Richmond Danso-Danquah, Boshi Huang, Yan Zhang, Faik N. Musayev, Osheiza Abdulmalik, Martin K. Safo

**Affiliations:** 1 Department of Medicinal Chemistry and The Institute for Structural Biology, Drug Discovery and Development, School of Pharmacy, Virginia Commonwealth University, Richmond, VA 23298, USA; alhashimirt@vcu.edu (R.T.A.); msghatge@vcu.edu (M.S.G.); donkora@vcu.edu (A.K.D.); alramadhanid@vcu.edu (D.A.); rddanso@gmail.com (R.D.-D.); bhuang2@vcu.edu (B.H.); yzhang2@vcu.edu (Y.Z.); fmoussae@vcu.edu (F.N.M.); 2 Vertex Pharmaceutical Incorporated, Boston, MA 02210, USA; deshpandetm@gmail.com; 3 Division of Hematology, The Children’s Hospital of Philadelphia, Philadelphia, PA 19104, USA; Nancyaa@sas.upenn.edu (N.A.); abdulmalik@email.chop.edu (O.A.)

**Keywords:** hemoglobin, sickle cell disease, antisickling, nitric oxide, oxygen equilibrium curve

## Abstract

5-hydroxyfurfural (5HMF), an allosteric effector of hemoglobin (Hb) with an ability to increase Hb affinity for oxygen has been studied extensively for its antisickling effect in vitro and in vivo, and in humans for the treatment of sickle cell disease (SCD). One of the downstream pathophysiologies of SCD is nitric oxide (NO) deficiency, therefore increasing NO (bio)availability is known to mitigate the severity of SCD symptoms. We report the synthesis of an NO-releasing prodrug of 5HMF (5HMF-NO), which in vivo, is expected to be bio-transformed into 5HMF and NO, with concomitant therapeutic activities. In vitro studies showed that when incubated with whole blood, 5HMF-NO releases NO, as anticipated. When incubated with sickle blood, 5HMF-NO formed Schiff base adduct with Hb, increased Hb affinity for oxygen, and prevented hypoxia-induced erythrocyte sickling, which at 1 mM concentration were 16%, 10% and 27%, respectively, compared to 21%, 18% and 21% for 5HMF. Crystal structures of 5HMF-NO with Hb showed 5HMF-NO bound to unliganded (deoxygenated) Hb, while the hydrolyzed product, 5HMF bound to liganded (carbonmonoxy-ligated) Hb. Our findings from this proof-of-concept study suggest that the incorporation of NO donor group to 5HMF and analogous molecules could be a novel beneficial strategy to treat SCD and warrants further detailed in vivo studies.

## 1. Introduction

Sickle cell disease (SCD) occurs as a result of a single point mutation of βGlu6 in normal hemoglobin (HbA) to βVal6 in sickle hemoglobin (HbS), which leads to stereospecific interactions between deoxygenated HbS (deoxyHbS) molecules to form polymers, causing red blood cells (RBCs) to deform into rigid sickled cells [1,2,3,4]. This primary pathophysiology leads to a cascade of secondary adverse effects that include occlusion of rigid sickle RBCs in capillaries and small blood vessels and obstructing blood flow (vaso-occlusion), hemolytic anemia, hypoxia, stroke, and organ damage [5,6]. Sickled RBCs are also very fragile, leading to hemolysis and consequent release of free hemoglobin (Hb) into blood circulation. The free plasma Hb is a potent nitric oxide (NO) scavenger, and not only leads to NO deficiency but also results in the formation of reactive oxygen species, such as superoxide and peroxide radicals [7,8,9,10]. Furthermore, the enzyme arginase released from the hemolyzed RBCs depletes plasma arginine levels. Arginine is an important source for NO production in the body by the enzyme NO synthase [11,12]. The ensuing deficiency of NO from the blood plasma leads to hypertension, inflammation; all common pathological conditions of SCD [13,14]. Four drugs, hydroxyurea, [15,16] Endari, [17] Crizanlizumab, [18] and Voxelotor [19,20,21,22] are currently approved for the treatment of SCD. While hydroxyurea has been used for over two decades, the other drugs were only recently approved. However, the inherent complexity of SCD suggests that none of the individual drugs are likely to universally be effective across patient populations, therefore the need remains for newer therapeutic options, particularly those that may incorporate more than one mechanism of action.

Hb is responsible for ensuring oxygen (O_2_) distribution in the body by binding and transporting O_2_ from the lungs to tissues. It carries out this function by equilibrating between the high-O_2_-affinity relaxed state (R-state) and the low-O_2_-affinity tense state (T-state). The relaxed or R-state is an ensemble of relaxed quaternary hemoglobin, which includes the classical R-state, R2-state, RR2-state, R3-state, etc. [23]. The O_2_ affinity of Hb is denoted as P_50_, the partial pressure of oxygen in mmHg, at which 50% of Hb is saturated with oxygen. Stabilization of deoxyHb or T-state Hb by allosteric effectors of Hb leads to an increase in the P_50_ (decrease Hb affinity for oxygen) or shifts the oxygen equilibrium curve (OEC) to the right, which can be potentially useful for increasing tissue oxygenation [23]. On the other hand, stabilization of the R-state and/or destabilization of the T-state leads to a decrease in the P_50_ (increase Hb affinity for oxygen) or shifts the OEC to the left [23]. Such compounds are potential antisickling agents since high-O_2_-affinity HbS does not polymerize [23]. A number of aromatic aldehydes that bind and increase Hb affinity for oxygen have been studied by our group for their potential therapeutic application in SCD treatment [24,25,26,27,28,29,30]. These aldehydes form Schiff base adducts with the N-terminal αVal1 amines at the α-cleft of Hb to stabilize the R-state Hb and prevent hypoxia-induced HbS polymerization [24,25,26,27,28,29,30]. Voxelotor, is an example of aromatic aldehyde that was recently approved by the FDA in 2019 for the treatment of SCD [19,20,21,22]. Another example is 5-hydroxymethylfurfural (5HMF), a natural aromatic aldehyde that was studied in the clinic prior to Voxelotor discovery [25,26,27,31,32]. In vitro studies show that 5HMF formed an adduct with Hb (modified Hb), increased Hb affinity for oxygen, decreased HbS polymerization and RBC sickling, reduced sickle cell (SS) mechanical fragility, and reduced RBC hemolysis [25,26,27,31,32]. Under severe hypoxic conditions, 5HMF also significantly reduced the formation of sickled cells and prolonged the survival time of a SCD mouse model when administered orally [26]. 5HMF underwent phase I clinical trials for the treatment of SCD but failed to pass beyond Phase II trials, due in part to the unblinding of the study result and suboptimal pharmacokinetic properties [31,32].

Another treatment modality to mitigate SCD severity is to target the secondary pathophysiology of the disease with NO therapy. NO is an endothelium-derived relaxing factor (EDRF) that is highly involved in blood vessel dilatation and smooth muscle relaxation [33,34,35]. NO also modulates inflammation by inhibiting vascular remodeling [36]. NO is generated from L-arginine by NO synthase that is primarily found in the vascular endothelium, and from nitrite/nitrate by Hb and in the RBCs [37,38,39]. Deficiency of NO in blood plasma is one of the common pathological conditions in SCD that potentiates hemolysis, inflammation, vaso-occlusion (VOC), hypertension, oxidative stress, and tissue damage [13,40]. Inhaled NO therapy has been reported clinically to be useful in SCD patients [10,14,41,42]. In addition to inhalation of NO, long-lived circulating NO-releasing nanoparticles (NO-nps) and several NO-donor compounds, e.g., hydroxyurea, nitroglycerine, amyl nitrite, isosorbide dinitrate, NO-NSAIDS, nitrosothiols, nicorandil, sinitrodil, have been shown to have positive vasodilatory, anti-inflammatory and anti-hypertensive effects in SCD patients [43,44,45,46,47].

**Figure 1:**
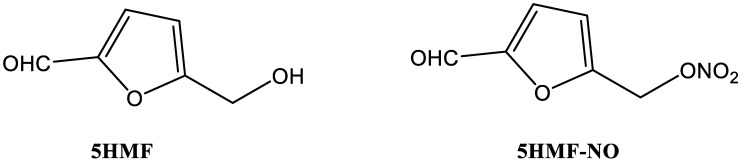
Structures of 5HMF and its NO-releasing derivative.

Based on the positive results of 5HMF as an antisickling agent and the potential benefits of providing exogenous NO in SCD patients, we decided to introduce NO releasing moiety to 5HMF with the aim of providing a novel approach for targeting several pathologies of the disease. A NO releasing moiety was added to the methylhydroxyl of 5HMF to form a nitrate ester of 5HMF (5HMF-NO; Figure 1). 5HMF-NO was further evaluated in vitro for its ability to release NO, form adduct with Hb (Hb modification), increase Hb affinity for oxygen, prevent hypoxia-induced sickling, and finally for its atomic interaction with Hb.

## 2. Materials and Method

### 2.1. Study Approvals

At Virginia Commonwealth University (VCU), normal whole blood (AA) was collected from adult donors (>18 years) after informed consent, in accordance with regulations of the IRB for Protection of Human Subjects (IRB #HM1) by the Institutional Review Board at VCU. At the Children’s Hospital of Philadelphia (CHOP), leftover blood samples from individuals with homozygous sickle cell (SS) who had not been recently transfused, were obtained and utilized based on an approved IRB protocol (IRB# 11-008151) by the Institutional Review Board, with informed consent. All experimental protocols and methods were performed in accordance with institutional (VCU and CHOP) regulations. Hemoglobin for crystallographic studies was prepared as previously published [48,49].

### 2.2. Chemistry

All reagents used in the syntheses and functional assays were purchased from Sigma-Aldrich corporation and Fisher Scientific Company and used without purification. ^1^H-NMR and ^13^C-NMR spectra were obtained on a Brucker 400 MHz spectrometer using tetramethylsilane (TMS) as an internal standard. Peak positions are given in parts per million (δ). Column chromatography was performed on silica gel (grade 60 mesh; Bodman Industries, Aston, PA, USA). Routine thin-layer chromatography (TLC) was performed on silica gel GHIF plates (250 μm, 2.5 × 10 cm; Analtech Inc., Newark, DE, USA). Elemental analysis was performed by Atlantic Microlab Inc. (Norcross, GA, USA) for the elements indicated and the results are within 0.4% of calculated values. Infrared spectra were obtained on a Thermo Nicolet iS10 FT-IR. Purity of the compounds was determined by HPLC using a Varian Microsorb 100-5 C18 column (250 × 4.6 mm), using Prostar 325 UV-Vis (254 nm) as the detector.

#### Synthesis of 5-Nitrooxymethyl Furfural (5HMF-NO)

5HMF-NO was prepared according to literature procedure for a similar compound [50]. Trifluoroacetic anhydride (5.5 mL, 2 mmol) was added to a suspension of lithium nitrate (2.7 g, 2 mmol) in anhydrous CH_3_CN (30 mL) at 20 °C. The reaction mixture was stirred until clear (30 min) and cooled to 0 °C (ice-bath). Sodium carbonate (4.2 g, 2 mmol) and 5HMF (2.5 g, 1 mmol) were added together and the reaction mixture was allowed to stir for 7 h at 0 °C (ice-bath). The reaction mixture was poured into an ice-cold solution of saturated NaHCO_3_ and extracted with EtOAc (2 × 10 mL). The combined organic portion was washed with brine (10 mL), dried over Na_2_SO_4_ and evaporated under reduced pressure to yield a crude product, which was purified by column chromatography (petroleum ether/EtOAc; 2:1) to afford 2.9 g (85%) of 5HMF-NO as a colorless viscous oil: IR (KBr, cm^−1^): 1678 (C = O), 1631, 1276 (NO_2_), 845 (ON); ^1^H-NMR (CDCl_3_): δ 5.48 (s, 2H, CH_2_), 6.73 (d, *J* = 3.5 Hz, 1H, ArH), 7.25 (d, *J* = 3.6, 1H, ArH), 9.68 (s, 1H, CHO); ^13^C-NMR (CDCl_3_): δ 177.9, 153.4, 151.5, 120.9, 114.5, 65.4. Anal. Calcd for (C_6_H_5_NO_5_) C, 42.12; H, 2.95; N, 8.19. Found: C, 42.02; H, 2.86; N, 8.26. The purity of the compound was checked by HPLC and found to be 98.8% pure.

### 2.3. Study of NO Release from 5HMF-NO in Whole Blood

The ability of 5HMF-NO to release the bound NO when incubated with whole blood was tested in a time-dependent manner using Griess assay as reported in the literature [51]. In this assay, whole blood from normal healthy individuals (adjusted to a hematocrit level of 30%) was mixed in 96-well plate (in duplicate or triplicate) with 5HMF-NO or 5HMF (control) to a final concentration of 1 mM and incubated in a shaker at 37 °C. Aliquots of blood were withdrawn at 0.5, 1, 2, 4, 6 and 18 h, and subsequently centrifuged (in a microcentrifuge) to separate out the plasma. To 50 µL of the separated plasma, 2.5 µL *Aspergillus* nitrate reductase (0.05 U), 0.25 µL FAD (5 µM) and 0.25 µL NADPH (0.1 mM) were added to convert nitrate to nitrite. 197 µL buffer (50 mM HEPES, PH 7.5) was added to adjust the volume to 250 µL. Next, the tubes were incubated at room temperature for 2 h. Then, 2.5 µL lactate dehydrogenase (2 mg/mL) and 25 µL Na pyruvate (100 mM stock) were added to consume leftover NADPH, and the tubes further incubated at room temperature for another 10 to 15 min. 50 µL of freshly mixed Griess reagent (Thermo Scientific, Waltham, MA, USA) was added to 125 µL of the reaction mixture, and the reaction volume was made to 1.5 mL distilled water. The tubes were then incubated in the dark for 30 min. The absorbance of the samples was read at 548 nm. Solutions of 0–100 µM sodium nitrite control solutions were used to generate the standard curve by a nitrite absorbance versus concentration under the same experimental conditions. Nitrite ion released by 5HMF-NO was calculated from the standard nitrite concentration curve.

### 2.4. In Vitro Hb Modification, Oxygen Equilibrium and Antisickling Studies Using Human Homozygous Sickle Cell (SS) Blood

5HMF-NO and the control 5HMF were studied for their abilities to modify Hb, increase Hb affinity for oxygen and inhibit hypoxia-induced RBC sickling (RBC morphology study) as previously described [26,27,30]. Briefly, SS blood (hematocrit of 20%) suspensions were incubated under air in the absence or presence of 1, 2 or 5 mM concentration of test compound at 37 °C for 1 h. Next, the suspensions were incubated under hypoxic condition (2.5% O_2_/97.5% N_2_) at 37 °C for 2 h. Aliquot samples were fixed with 2% glutaraldehyde solution without exposure to air, and then subjected to microscopic morphological analysis. To determine whether 5HMF-NO retained the parent 5HMF’s ability to form adduct and modify Hb, hemolysates from the above antisickling studies were subjected to a cation-exchange HPLC (Hitachi D-7000 Series, Hitachi Instruments, Inc., San Jose, CA, USA) using a weak cation-exchange column (Poly CAT A: 30 mm × 4.6 mm, Poly LC, Inc., Columbia, MD, USA). Finally, for the oxygen equilibrium studies, 100 µL aliquot samples from the above clarified lysate were added to 4 mL of 0.1 M potassium phosphate buffer, pH 7.0, in cuvettes and subjected to hemoximetry analysis using a Hemox^TM^ Analyzer (TCS Scientific Corp., New Hope, PA, USA) to determine the P_50_ values.

### 2.5. Crystallographic Study of Hb Co-Crystallized with 5HMF-NO

X-ray crystallography was performed to study the atomic interactions of 5HMF-NO with Hb, following the previously reported procedure [27,28]. For the crystallization experiment, 40 mg/mL of Hb was incubated with 20 molar excess of 5HMF-NO, and then evacuated for ~1 h to make the deoxyHb-compound complex solution. Next, sodium cyanoborohydride (NaCNBH_4_) in 10 molar excess of Hb was added to reduce the reversible Schiff base adduct formed between the amino group of Hb and the aldehyde of 5HMF-NO to the corresponding irreversible alkylamine covalent bond. Subsequent crystallization of the deoxyHb-compound complex in test tubes using 3.2–3.6 M sulfate/phosphate precipitant pH 6.5, and ferrous citrate (50 μL) in a glovebox under nitrogen atmosphere led to formation of two different crystal forms [27,28]. The crystals, corresponding to T-state and R2-state crystals were used for X-ray diffraction data collection at 100K using Rigaku MicroMax™ 007HF X-ray Generator, Eiger R 4M Detector and Oxford Cobra Cryo-system (The Woodlands, TX, USA). The crystals were first washed in a cryoprotectant solution containing 50 µL mother liquor and 10–12 µL glycerol prior to X-ray diffraction. The collected data sets were processed with the CrysAlisPro software program (Rigaku) and the *CCP*4 suite of programs [52]. The T-state and R2-state crystal structures were refined using the Phenix program with the previously reported isomorphous T-state and R2-state crystal structures (PDB ID: 2HHB and 1QXE), respectively, as starting models. Model building and correction were carried out using COOT [53,54,55]. The final Rfactor/Rfree of the T-state and R2-state structures are 15.1/19.1 and 18.6/22.8%, respectively. 

## 3. Results and Discussion

### 3.1. Chemistry

**Scheme 1:**
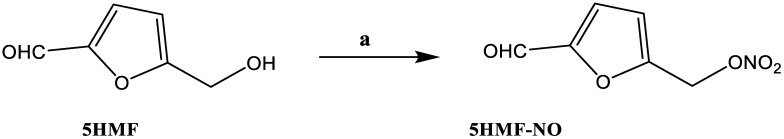
**5**HMF-NO. (**a**) LiNO_3_, TFAA, Na_2_CO_3_, anhydrous CH_3_CN, 0 °C, 7 h.

In this study, we incorporated an NO-donor group onto the previously studied antisickling compound, 5HMF, to give the nitrate ester prodrug 5HMF-NO, as shown in Scheme 1. Briefly, 5HMF was nitrated directly using lithium nitrate and trifluoroacetic anhydride under basic conditions at zero degrees Celsius to give 5HMF-NO in 98.8% yield (Figure S1 in Supplementary Materials).

### 3.2. 5HMF-NO Is Capable of Releasing NO

**Figure 2:**
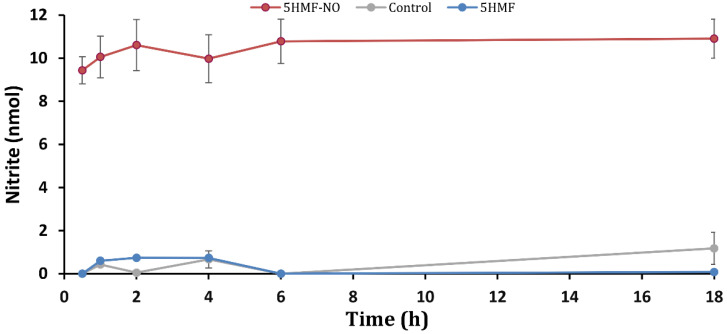
**Time-dependent NO release from 5HMF-NO.** NO release (in the form of nitrite) by 5HMF-NO (red), 5HMF (cyan), and no test compound (grey). The results are the mean values ± SD for biological duplicate or triplicate experiments. All compounds were solubilized in 2% DMSO. Control experiment without test compound also included 2% DMSO. Final test compound concentration was 1 mM.

The key to the effectiveness of 5HMF-NO is its ability to release NO and the parent 5HMF for their respective beneficial effects. Griess assay was therefore used to measure the NO release from 5HMF-NO using human whole blood. Aliquots of the compounds incubated with the blood at 37 °C were withdrawn at various time points (0.5, 1, 2, 4, 6 and 18 h), and plasma obtained from these samples was added to the Griess reagent to detect the released NO in the form of nitrite ion (NO_2_^−^). The nitrite release (nmol) was calculated from a standard NaNO_2_ curve by extrapolating the absorbance (548 nm) of the Griess reagent-nitrite azo dye complex against the concentration of NaNO_2_. Results, expressed as the concentration of nitrite versus time (Table 1 and Figure 2) show that 5HMF-NO was able to release nitrite over the course of the experiment, while, the control group (5HMF or absence of compound) exhibited no formation of nitrite. There is a quick release of 9.4 nmol nitrite at 0.5 h, which slightly increased to about 11 nmol at two hours, and then sustained throughout the 18-h experiment. We expect a similar release of 5HMF and NO in vivo, although the amount and mode of NO release could be different. Based on the previously proposed mechanism of biotransformation of NO-releasing prodrugs from nitrate ester groups, [46,51,56] we expect the release of the NO to involve both enzymatic action and chemical hydrolysis. A potential pharmacologic disadvantage of 5HMF-NO is that its use could lead to clinical nitrate tolerance development, especially if the NO-release is dependent on metabolic activation that decreases in efficiency upon continued use [46,57].

The NO release in the form of nitrite (nmol) was calculated from a standard NaNO_2_ curve by extrapolating the absorbance (548 nm) of the Griess reagent-nitrite azo dye complex. The results are the mean values ± SD for biological duplicate or triplicate experiments. All compounds were solubilized in 2% DMSO. The final test compound concentration is 1 mM. The control experiments without a test compound also included 2% DMSO.

### 3.3. 5HMF-NO Forms Adduct with Hb to Increase the Affinity of Hb for Oxygen and Prevent Hypoxia-Induced RBC Sickling

**Figure 3:**
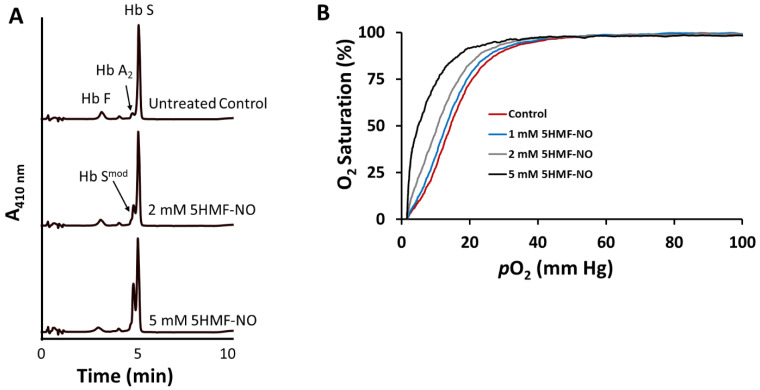
**Representative figures of Hb modification and OEC shift by 5HMF-NO in sickle blood:** (**A**) Cation exchange HPLC chromatograms showing a dose-dependent Hb modification; (**B**) OEC curves demonstrating a doses-dependent left-shift in oxygen affinity study.

**Figure 4:**
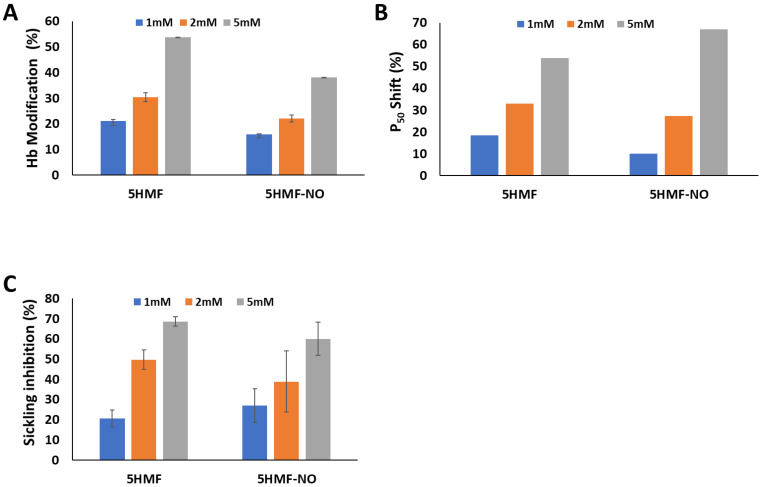
**Functional/biological effects of 5HMF-NO in sickle blood.** Hb modification, oxygen equilibrium and antisickling studies were conducted with SS blood suspensions (hematocrit of 20%) incubated with 1, 2 and 5 mM of 5HMF or 5HMF-NO. The final concentration of DMSO was <2% in all samples, including in control samples. The results are the mean values ± SD for two or three biological replicate experiments for the Hb modification and antisickling studies, and single measurement for the OEC study. The differences between the 5HMF and the 5HMF-NO groups were not statistically significant (*p* values of 0.2, 0.66, and 0.98, respectively). (**A**) Concentration-dependent modification of Hb S; (**B**) Concentration-dependent P_50_ shift of Hb S; (**C**) Concentration-dependent inhibition of SS cell sickling under hypoxia.

This study was conducted to determine the extent of Hb modification and antisickling activities of 5HMF-NO using SS blood and to correlate the result with its allosteric activity of modulating Hb oxygen binding properties. 5HMF-NO and the positive control 5HMF were dose-dependently (1, 2, and 5 mM) incubated with whole SS blood suspensions (hematocrit of 20%) under hypoxic conditions (2.5% O_2_/97.5% N_2_) at 37 °C for 2 h to form adduct with Hb. Aliquot blood samples were drawn and used for assessing RBC morphology, Hb affinity for oxygen and Hb adduct formation as previously described [26,27,29,30]. The results summarized in Table 2 and Figure 3 and Figure 4 show dose-dependent effects for all three pharmacodynamic parameters, and expectedly show direct correlation among them. 5HMF-NO modified Hb by 16%, 21% and 38% at 1 mM, 2 mM and 5 mM, respectively (compared to 21%, 31% and 54% by 5HMF), increase Hb affinity for oxygen (P_50_-shift) by 10%, 27%, and 67% (compared to 18%, 33%, and 54% by 5HMF), translating into RBC sickling inhibition of 27%, 39% and 60% (compared to 21%, 50% and 69% by 5HMF). It is clear that 5HMF-NO maintains the pharmacodynamic activities of 5HMF.

The results are the mean values ±SD for two or three biological replicate experiments for the Hb modification and antisickling studies, and single measurement for the OEC study. The differences between the 5HMF and the 5HMF-NO groups were not statistically significant (*p* values of 0.2, 0.66, and 0.98, respectively). All compounds were solubilized in DMSO (2%). The control experiments without a test compound also contain 2% DMSO.

ΔP_50_ (%) = P_50_ of lysates from untreated cells − P_50_ of lysates from treated cells × 100

P_50_ of lysates from untreated cells

### 3.4. 5HMF-NO When Co-Crystallized with Hb Binds to the T-State as 5HMF-NO and R2-State Hb as 5HMF

An X-ray crystallographic study was performed to help gain insight into the mechanism of action of 5HMF-NO, and detailed crystallographic data for the reported structures are shown in Table 3. As previously reported for several aromatic aldehydes, [27,28] deoxyHb when co-crystallized with 5HMF-NO resulted in two different crystal forms: dark purple rectangular-shaped T-state crystals that appeared within 2–4 days, followed by reddish trigonal-shaped R2-state crystals that appeared about four weeks later. Expectedly, the crystal parameters and eventually the corresponding structures are isomorphous to T-state (PDB 2HHB) and R2-state (PDB 1BBB) structures, respectively (Table 3). Aromatic aldehydes are known to preferentially bind to the relaxed R2-state conformation to increase Hb affinity for oxygen [23,27,28,58].

**Figure 5:**
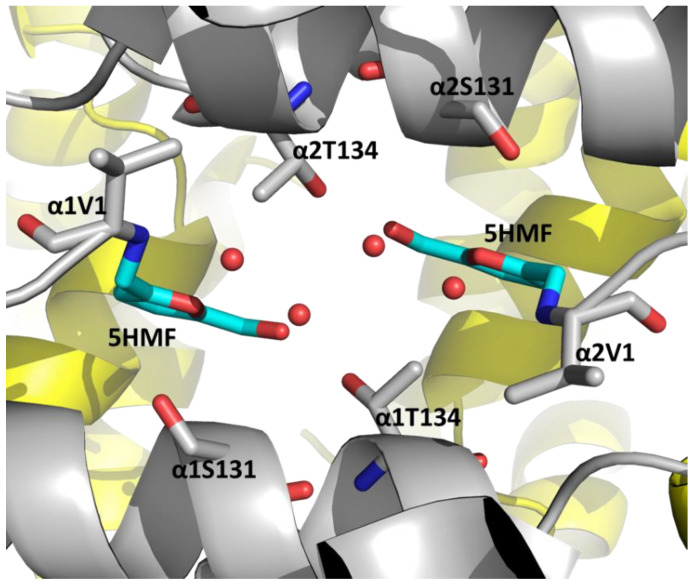
**Binding of a pair of 5HMF molecules (cyan sticks) at the α-cleft of R2-state Hb.** The α-chain (α1- and α2-subunits) colored in grey and β-chain (β1- and β2-subunits) colored in yellow. Water molecules are red spheres.

**Figure 6:**
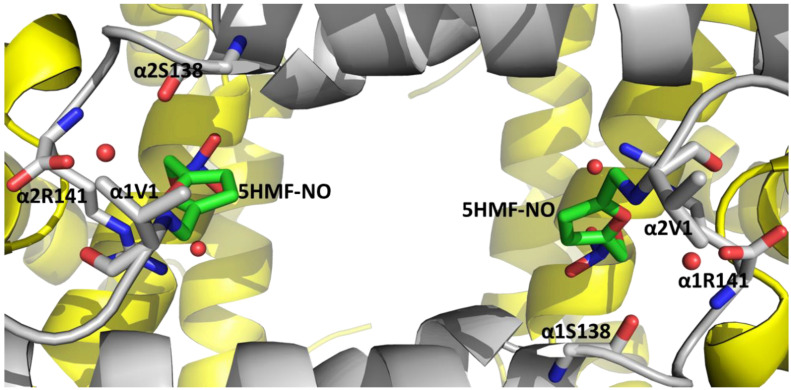
**Binding of a pair of 5HMF-NO molecules (green sticks) at the α-cleft of T-state Hb.** The α-chain (α_1_- and α_2_-subunits) colored in grey and β-chain (β_1_- and β_2_-subunits) colored in yellow. Water molecules are red spheres.

The T-state Hb or R2-state Hb structure showed two compounds bound at the α-cleft; the molecules forming Schiff base interactions with αVal1 amines of both α1- and α2-subunits in a symmetry-related fashion. Interestingly, while the T-state Hb binds the prodrug 5HMF-NO, the R2-state Hb binds the hydrolyzed product 5HMF (Figure 5 and Figure 6). It is clear that hydrolysis of the nitrate ester might have occurred during crystallization to release some 5HMF, which then bound to deoxygenated Hb. Similar chemical hydrolysis of the organic molecule nitrate esters to release NO, and crystallization has been reported [51]. Expectedly, the T-state Hb structure lacks oxygen at the hemes, while the R2-state Hb structure shows bound oxygen atoms at all four hemes, presumably from residual or slow diffusion of oxygen into the crystallization tube, as previously reported [27,28]. The root mean square deviation (Rmsd) between the R2-state Hb complex structure and the previously reported liganded R2-state Hb structure (PDB code IBBB) [59] or the T-state Hb complex structure and the previously reported unliganded T-state Hb structure (PDB code 2HHB) [60] is ~0.4 Å, suggesting that binding of the molecules to the respective quaternary structures does not induce any significant conformational changes.

The bound 5HMF molecules in the R2-state Hb structure is similar to the previously reported R2-state Hb structure in complex with 5HMF (PDB code 1QXE) [27]. In addition to the Schiff base interaction with the αVal1 amine of the α-subunits, 5HMF makes several intra- and inter-subunit protein interactions. At the α1-subunit binding site, the furan ring oxygen and the methylhydroxyl moiety of 5HMF engage in intra-subunit hydrogen-bond interactions with α1Ser131 OG (3.2 Å) and α1Thr134 OG1 (2.6 Å), respectively (Figure 5). Similar symmetry-related interactions by the second molecule are observed at the α2-subunit. Additionally, the two bound 5HMF molecules are joined together, and to the protein by a network of several inter-subunit and intra-subunit water-mediated hydrogen-bond interactions, through the methylhydroxyl and the furan ring oxygen moieties (Figure 5). These interactions, as previously noted, [27] serve to tie the two α-subunits together, and expected to stabilize the relaxed state Hb, explaining the compound’s ability to increase Hb affinity for oxygen with a concomitant antisickling effect.

Unlike the R2-state Hb structure that binds 5HMF, the T-state Hb interestingly binds 5HMF-NO. In addition to the Schiff base interaction by 5HMF-NO with the α2-subunit α2Val1 amine, the furan oxygen forms inter-subunit water-mediated hydrogen-bond interactions with the amide oxygen of α1Ser138 and the carboxyl atom of α1Arg141 (Figure 6). The ester oxygen atom and one of the nitro oxygen atoms of 5HMF-NO make inter-subunit hydrogen-bond interactions with the guanidinium group of α1Arg141 (3.2 Å and 3.3 Å, respectively). There is also an inter-subunit water-mediated hydrogen-bond interaction between the ester oxygen atom and the guanidinium group of α1Arg141. The nitro group oxygen also makes direct inter-subunit hydrogen-bond interaction with the amide of α1Thr137 (3.4 Å). The second 5HMF-NO molecule forms a Schiff-base interaction with the α1-subunit α1Val1 amine and makes similar symmetry-related interactions with the protein as described above for the first 5HMF-NO molecule at the α2Val1 binding site, although in some instances with slightly different bond distances. The interactions by 5HMF-NO are expected to stabilize the T-state Hb and restrict the transition to the R-state.

It is interesting that, in the R2 structure, the furan oxygen of 5HMF faces the central water cavity to make an intra-subunit hydrogen-bond interaction with αSer131 OG, as well as water-mediated interactions with the protein, while in the T-state the furan ring of 5HMF-NO has rotated ~180° such that the oxygen makes an inter-subunit interaction with the guanidinium group of αArg141. Furthermore, notably, unlike the R2-state structure where 5HMF mediates both inter-subunit and intra-subunit interactions with the protein, in the T-state structure, all the hydrogen-bond interactions are inter-subunit in nature. The T ↔ R allosteric transition is known to lead to significant rearrangement of the α1-subunit position relative to the α2-subunit, resulting in a much larger central water cavity in the T-state Hb compared to the R2-state Hb. Consquently, the two 5HMF-NO moleules in the T structure are seperated by 11.7 Å without any contact between the molecules. In contrast, the two bound 5HMF molecules in the R2 structure are only seperated by 4.3 Å, interacting together through several water-mediated hydrogen-bond interactions.

It is clear that unlike unlike 5HMF that binds to Hb and stabilizes the R2-state Hb, the binding of 5HMF-NO leads to the stabilization of T-state Hb. As previously noted, [23,58,61] the apparent stabilization of T-state Hb by 5HMF-NO is expected to lead to a decrease in Hb affinity for oxygen, which is clearly opposite to our observation of increased Hb affinity for oxygen when 5HMF-NO was tested with whole blood. It thus appears that in whole blood, there is significant hydrolysis of the ester nitrate to form 5HMF. It is assumed that in vivo, and particularly in the RBC, 5HMF-NO will exist predominantly as 5HMF, otherwise any significant amount of unhydrolyzed 5HMF-NO in the RBC could have a negative impact on sickling.

## 4. Conclusions

5HMF has previously been studied in the clinic for the treatment of SCD as a result of its ability to stabilize the high-O_2_-affinity R-state Hb, and increase the concentration of the non-polymerizing oxygenated HbS [26,27]. We have synthesized NO-releasing prodrug of 5HMF (5HMF-NO) that is expected to be bio-transformed into 5HMF and NO in vivo and exhibit therapeutic activities. It is expected that controlled delivery of therapeutic quantities of NO—concurrently with 5HMF’s primary antipolymerization properties—will ameliorate both primary and secondary pathophysiologic effects, including RBC sickling and hemolysis, inflammation, oxidative stress, painful VOC, and chronic organ damage. Our in vitro functional and biological analyses showed that 5HMF-NO is capable of hydrolyzing to release NO and 5HMF to increase the affinity of Hb for oxygen with a concomitant antisickling effect. Although, not studied for its pharmacologic effect, we expect the released NO to have a beneficial effect, and future studies will test this hypothesis.

## Figures and Tables

**Table 1:** NO release from 5HMF-NO in human whole blood.

	NO_2_^−^ Concentration (nmol)					
Compound	0.5 h	1 h	2 h	4 h	6 h	18 h
**5HMF-NO**	9.44 ± 0.63	10.1 ± 0.97	10.6 ± 1.19	9.98 ± 1.11	10.8 ± 1.03	10.9 ± 0.91
**5HMF**	0.00 ± 0.00	0.59 ± 0.01	0.73 ± 0.01	0.73 ± 0.01	0.30 ± 0.02	0.079 ± 0.00
**Control**	0.00 ± 0.04	0.42 ± 0.00	0.04 ± 0.28	0.66 ± 0.00	0.00 ± 0.52	1.18 ± 0.52

**Table 2:** Hemoglobin modification, change in oxygen equilibrium, and antisickling studies of 5HMF and 5HMF-NO using human sickle blood.

Compound	% Functional/Biological Effect								
	Hb Modification			Sickling Inhibition			ΔP_50_		
	1 mM	2 mM	5 mM	1 mM	2 mM	5 mM	1 mM	2 mM	5 mM
**5HMF-NO**	15.9 ± 0.2	20.7 ± 1.3	38.1 ± 0.6	27.0 ± 8.4	38.9 ± 15.1	59.9 ± 8.2	10.0	27.2	66.9
**5HMF**	21.1 ± 0.6	30.9 ± 1.7	53.7 ± 0.1	20.6 ± 4.2	49.7 ± 4.8	68.5 ± 2.3	18.4	33.0	53.9

**Table 3:** Crystallographic data and refinement statistics for unliganded (T-state) Hb in complex with 5HMF-NO and liganded (R2-state) Hb in complex with 5HMF.

Data Collection Statistics	5HMF-NO (T-State)	Hydrolyzed 5HMF (R2-State)
PDB ID	7UD7	7UD8
Space group	P2_1_	P3_2_21
Cell dimensions (Ǻ)	62.34, 82.03, 53.45; 99.49	92.23, 92.23, 144.07
Tetramer/AU	1	1
Resolution (Ǻ)	29.92–1.80 (1.86–1.80)	28.39–1.80 (1.84–1.80)
Measured reflections	18,7917	71,6379 (20,836)
Unique reflections	46,300 (4367)	66,309 (3896)
Redundancy	4.06 (3.81)	10.8 (5.3)
I/σI	25.9 (10.8)	28.8 (2.0)
Completeness (%)	94.1 (89.4)	100 (99.7)
R_merge_ (%) ^a^	3.9 (8.2)	5.5 (72.7)
**Structure refinement**		
Resolution limit (Ǻ)	26.36–1.80 (1.86–1.80)	28.39–1.80 (1.86–1.80)
No. of reflections	46,299 (4366)	66,260 (6521)
R_work_ (%)	15.1 (18.9)	18.6 (24.1)
R_free_ (%) ^b^	19.1 (24.4)	22.8 (26.9)
Rmsd geometry		
Bond lengths (Ǻ)	0.007	0.026
Bond angles (°)	1.37°	2.01°
Dihedral angles (%)		
Most favored regions	98.2	98.1
Allowed regions	1.6	1.6
Average B-factors (Å^2^)		
All atoms	15.6	27.3
Protein alone	13.84	26.2
Ligands	13.74	24.5
Water	26.18	35.4

^a^ Rmerge = Ʃ_hkl_Ʃ_i_/I_hkli_ − <I_hkli_>/Ʃ_hkl_Ʃ_i_<I_hkli_>. ^b^ Rfree was calculated with 5% excluded reflection from the refinement.

## Data Availability

The atomic coordinates and the structure factor of 5HMF-NO and 5HMF with Hb are deposited in the RCSB Protein Databank (https://www.rcsb.org/, accessed on 3 April 2022) with entries 7UD7 and 7UD8, respectively.

## Supplementary Materials

The following supporting information can be downloaded at: https://www.mdpi.com/article/10.3390/biom12050696/s1, Figure S1: HPLC Chromatogram of 5HMF-NO.

## Abbreviations

Hb, hemoglobin; HbS, sickle hemoglobin; OEC, oxygen equilibrium curve; RBC, red blood cell; SCD, sickle cell disease; deoxyHb, deoxygenated hemoglobin; NO, nitric oxide; R-state or R2-state, relaxed state; T-state, tense state; 5HMF, 5-hydroxymethylfurfural; PO2, partial pressure; SO2, oxygen saturation; O2, oxygen.
