# Supplementary figures and images for: Design, Synthesis, and Antisickling Investigation of a Nitric Oxide-Releasing Prodrug of 5HMF for the Treatment of Sickle Cell Disease
Source: Biomolecules. 2022 May 12;12(5):696. doi: 10.3390/biom12050696 (PMC9138457; doi:10.3390/biom12050696)

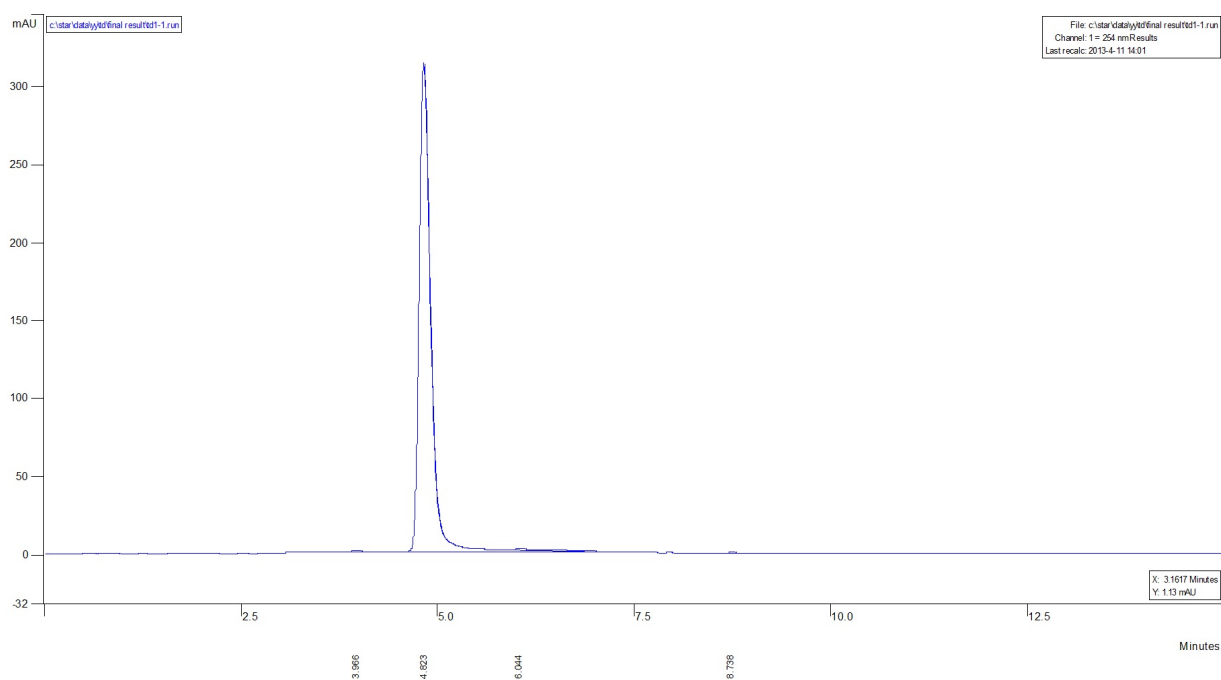

Figure S1: HPLC Chromatogram of 5HMF-NO

Supplement: Supplementary file 1 [file biomolecules-12-00696-s001.zip › biomolecules-1692198-supplementary.pdf]
